# Architecture of divergent flagellar promoters controlled by CtrA in *Rhodobacter sphaeroides*

**DOI:** 10.1186/s12866-018-1264-y

**Published:** 2018-10-10

**Authors:** Anet Rivera-Osorio, Aurora Osorio, Sebastian Poggio, Georges Dreyfus, Laura Camarena

**Affiliations:** 1 Instituto de Investigaciones Biomédicas, Universidad Nacional Autónoma de México, México City, México; 2 Instituto de Fisiología Celular, Universidad Nacional Autónoma de México, México City, México

**Keywords:** *Rhodobacter sphaeroides*, CtrA, Bacterial flagellum, Divergent promoters

## Abstract

**Background:**

*Rhodobacter sphaeroides* has two sets of flagellar genes, *fla1* and *fla2*, that are responsible for the synthesis of two different flagellar structures. The expression of the *fla2* genes is under control of CtrA. In several α-proteobacteria CtrA is also required for the expression of the flagellar genes, but the architecture of CtrA-dependent promoters has only been studied in detail in *Caulobacter crescentus*. In many cases the expression of *fla* genes originates from divergent promoters located a few base pairs apart, suggesting a particular arrangement of the *cis*-acting sites.

**Results:**

Here we characterized several control regions of the *R. sphaeroides fla2* genes and analyzed in detail two regions containing the divergent promoters *flgB2p*-*fliI2p*, and *fliL2p*-*fliF2p*. Binding sites for CtrA of these promoters were identified in silico and tested by site directed mutagenesis. We conclude that each one of these promoter regions has a particular arrangement, either a single CtrA binding site for activation of *fliL2p* and *fliF2p*, or two independent sites for activation of *flgB2p* and *fliI2p*. ChIP experiments confirmed that CtrA binds to the control region containing the *flgB2* and *fliI2* promoters, supporting the notion that CtrA directly controls the expression of the *fla2* genes. The *flgB* and *fliI* genes are syntenic and show a short intercistronic region in closely related bacterial species. We analyzed these regions and found that the arrangement of the CtrA binding sites varies considerably.

**Conclusions:**

The results in this work reveal the arrangement of the *fla2* divergent promoters showing that CtrA promotes transcriptional activation using more than a single architecture.

**Electronic supplementary material:**

The online version of this article (10.1186/s12866-018-1264-y) contains supplementary material, which is available to authorized users.

## Background

Many bacteria use rotating flagella for locomotion, the motor is embedded in the membrane and couples the influx of specific ions to the generation of rotational force. The bacterial flagellum can be divided in a basal body that includes the motor, a filament that acts as a propeller, and a universal joint also known as the hook which links the basal body and the filament. The basal body is the most complex structure that includes an axial rod and three or more rings, depending on the bacterial species. In *Escherichia coli* and *Salmonella enterica*, the basal body includes the MS-, P- and L-rings. Flagellar biogenesis requires the expression of more than 40 genes, and the assembly of this structure proceeds outwardly from proximal to distal end [1, 2]. In many bacterial species, the expression of the flagellar genes follows a hierarchical pattern with a variable number of tiers. Genes encoding the early components of the flagellar structure (basal body and export apparatus) are expressed under the control of a master activator protein placed at the top of the hierarchy. Frequently, along with the early genes, additional transcription factors are expressed that become active to transcribe the late genes in response to a signal that denotes that the early flagellar components have been assembled [3–5]. The flagellar gene expression regulatory hierarchies have been analyzed in different bacteria; in *E. coli* and *S. enterica* the transcriptional activator FlhD/FlhC is required to express the early flagellar genes (Class II) which encode the proteins required to form the basal body and the hook. The genes encoding the sigma factor FliA and its specific anti-sigma factor, FlgM also belong to this class. Once the basal body and the hook are assembled, FlgM is exported out of the cell and FliA associates with the core RNA polymerase to recognize class III promoters [5]. In other γ-proteobacteria (i.e. *Vibrio*, *Pseudomonas*), the flagellar gene expression hierarchy shows a different architecture and special features. In several species the sigma factor RpoN together with an activator protein promote the expression of the early flagellar genes, and the late genes are dependent on FliA. In these systems the expression of late flagellar genes also requires the export of FlgM [3]. Only a few examples of flagellar gene expression hierarchies have been reported for α proteobacteria; nonetheless, a substantial variation in the control networks is evident. For instance, in *Sinorhizobium meliloti* two members of the LuxR family, VisN and VisR [6], bring about the expression of *rem*, which encodes an OmpR-like transcriptional activator that promotes the expression of class II flagellar genes [7]. The expression of the class III genes that include the flagellin genes *flaA*, *flaB*, and *flaC* as well as several genes encoding chemotactic receptors are directly activated by the conserved transcriptional regulator CtrA [8]. The mechanism that turns on the expression of class III genes in *S. meliloti* is still unknown. In *Caulobacter crescentus*, the flagellar expression hierarchy has been thoroughly studied. In this case, CtrA activates the expression of the class II genes that encode the proteins of the MS ring and the flagellar export apparatus as well as the regulatory proteins RpoN and FlbD. Upon completion of the export apparatus and the MS-ring, FlbD is phosphorylated by an unknown kinase and together with the sigma factor RpoN promote transcription of class III/IV genes, whose products form the axial rod, the L- and P-rings, the hook and the flagellar filament [9, 10].

*Rhodobacter sphaeroides* is an α-proteobacterium with two flagellar systems of different phylogenetic origin [11]. Under the growth conditions commonly used in the laboratory, the *fla1* genes are expressed and direct the assembly of a single subpolar flagellum [12]. It has been shown that the *fla1* genes of *R. sphaeroides* were acquired by horizontal transfer. In contrast, the gene products of the vertically inherited *fla2* genes enable, under particular conditions, the synthesis of several polar flagella [11, 13]. The expression of the *fla1* genes follows a hierarchical expression pattern in which the early genes are expressed under the control of RpoN1 and the activator proteins FleQ and FleT whereas the late genes are dependent on FliA [14]. In contrast, expression of the *fla2* genes is dependent on the two-component system (TCS) formed by the hybrid histidine kinase, CckA, the histidine phosphotransferase, ChpT, and the response regulator CtrA [15]. The signal that fully activates CckA as a kinase has not been elucidated yet in this bacterium; therefore, the Fla2 system has been studied in strains that carry two mutations: one that inactivates the master regulator of the Fla1 system, and the other, a constitutive mutation in CckA. These strains, in consequence, have a Fla1^−^/Fla2^+^ phenotype [11, 15, 16].

The CckA/ChpT/CtrA TCS, is broadly distributed among α-proteobacteria. In some bacterial species this system is essential given that CtrA regulates the expression of genes that control the cell cycle; in contrast in many other bacteria, CtrA is not essential. In general, genes controlled by CtrA are enriched in certain functional categories, i.e. cell motility, signal transduction, and cell wall/membrane envelope biogenesis [17].

In *C. crescentus* the architecture of the CtrA promoters has been recently analyzed at genomic level by means of a global 5’ RACE protocol combined with a search for CtrA binding sites using a weight position matrix generated from ChIP experiments. From these studies it was observed that there are two classes of CtrA binding motifs, a full site represented by the sequence TTAA (N7) TTAA, and a half site (TTAA) [18]. Previously, a global analysis of the control regions of the CtrA-dependent genes, using MEME and BIOPROSPECTOR, also identified the sequence for the full site, the ungapped variant TTAACCAT, and the short motif TTAA [19]. In addition, it was reported that the CtrA binding sites involved in transcriptional activation are located near the − 35 promoter region. In contrast, in those promoters where CtrA acts as a repressor, the CtrA half binding motif overlaps with the − 10 promoter region [18]. The relevance of the TTAA (N7) TTAA motif for transcriptional activation has been proved by site directed mutagenesis [20].

For other bacteria, it was suggested that CtrA could have similar recognition sites based on the fact that related motifs have been identified upstream of genes controlled by CtrA [17, 21–24]. Supporting this idea, it has been observed that *ctrA* from *Rickettsia prowazekii* is partially functional in *C. crescentus*, and that *ctrA* from *C. crescentus* is functional in *Sinorhizobium meliloti* [25, 26].

In many species, the flagellar genes presumably controlled by CtrA are expressed as divergent transcriptional units, and in many cases, the control region encompasses less than 100 bp [27–29].

In this work we analyzed two divergent promoter regions carrying the *fla2* promoters *flgB2p*-*fliI2p* and *fliF2p*-*fliL2p*. The architecture of these regulatory regions was characterized using 5’-RACE, site directed mutagenesis and chromatin immunoprecipitation (ChIP) assay. From these analyses we conclude that CtrA directly activates the *fla2* promoters using more than a single architecture.

## Methods

### Strains, plasmids and oligonucleotides

All plasmids and bacterial strains used in this work are listed in Table 1. The oligonucleotides used in this work are listed in Table 2.

**Table 1:** Strains and plasmids used in this work

	Description	Reference
Strains		
*Rhodobacter sphaeroides*		
WS8N	wild-type; spontaneous Nal^R^	[52]
AM1	WS8N derivate, Fla2^+^, *ΔfleQ*::kan *cckA*_L391_	[16]
EA1	AM1 derivative, *ΔctrA*::*aadA*	[15]
AR1	AM1 derivative *ΔflgB2*-*fliI2*::Ω^Spc^	This work
*Escherichia coli*		
TOP10	Cloning strain	Invitrogen
S17–1	*recA endA thi hsdR* RP*4*–2-Tc::Mu::Tn*7*	[53]
Rosetta	Protein expression strain, Cm^R^	Novagen
Plasmids		
pTZ19R	Cloning vector Ap^R^	Fermentas
pCR2.1-TOPO	Cloning vector, Ap^R^	Invitrogen
pJQ200mp18	Mobilizable suicide vector; Gm^R^	[32]
pRK415	Expression vector for *R. sphaeroides*	[54]
pRK415/uidA	pRK415 carrying the *uidA*-*aadA* cassette	This work
pWM5	Vector source of the *uidA*-*aadA* cassette	[55]
pRK_flgB2p::uidA-aadA	pRK415/uidA carrying *flgB2p*	This work
pRK_flgE2p::uidA-aadA	pRK415/uidA carrying *flgE2p*	This work
pRK_fliF2p::uidA-aadA	pRK415/uidA carrying *fliF2p*	This work
pRK_fliI2p::uidA-aadA	pRK415/uidA carrying *fliI2p*	This work
pRK_fliL2p::uidA-aadA	pRK415/uidA carrying *fliL2p*	This work

**Table 2:** Oligonucleotides used in this work^a^

To obtain the strain AR1	
DinterB2for	TCTAGATGGAACTCCTTTCAACGAC
DinterI2rev	TCTAGAACGGTCGTCGTCTACGA
To obtain pBAD_ctrA	
CtrABADFwSac	GAGCTCATGAGAATACTGCTGGTGGA
CtrABADRvEco	GAATTCTCCAGCCCACCCTTCCCG
To clone the wild-type promoters	
*flgB2p* (336 bp)	
FlgBI5	GAGTCTGATATCCGGGCGTGTCGGCATTG
FlgBI2	GAATTCCACCCGGTCGCCCAGCGCGG
*flgE2p* (303 bp)	
pflgE2for	GAATTCCGGTGCGAAACAACAGACT
pflgE2rev	GAGCTCATTGGCCGACTGCGTGAT
*fliF2p* (331 bp)	
FliFL5	GAGCTCAGACCGAGCACGGCCAGGAA
FliFL2	GAATTCAGGCCCGACCAGGTGGCGTAG
*fliI2p* (product of 336 bp)	
FliBI3	GAATTCGATATCCGGGCGTGTCGGCATTG
FliBI6	GAGCTCCACCCGGTCGCCCAGCGCGG
*fliL2p* (331 bp)	
FliFL3	GAATTCAGACCGAGCACGGCCAGGAAC
FliFL6	GAGCTCAGGCCCGACCAGGTGGCGTAG
For the 5’ RACE experiments	
flgB2 RACE	CGCACCATCTCATCCTCGAGCGAG
N3’ flgB2 RACE	GACCGTGTTGCCGTTGGGCGAAG
flgE2 RACE	GATATCCAGGGCGCTCGCGGTCGAGA
N3’ flgE2 RACE	CATTCCTCCACCCGCCGTATGGTGTT
fliF2 RACE	CACCTCGTAGGCCGCGCCCTGCGCC
fliI2 RACE	GGGCAGGATCGCCACCTCGTGCGACGAA
N3’ fliI2 RACE	GCCGCAGAACCTCGCCGCCGAGGATG
fliL2 RACE	CCAAGGCTGATCACGATCGGGTCGA
N3’ fliL2 RACE	CGATCGGCACGAAGGCGATGTCGGGA
For site directed mutagenesis	
flgB2p −10	GTTTCACCAAGGCGCAAGGGCGATTCCTTTAG
flgB2p −13/−14	CACCAAGGCTTCGGGGCGATTC
flgB2p −17/−18	CAAGGCTTAAGGCGGATTCCTTTAGAAAG
flgB2p −23/−24	CTTAAGGGCGATTGGTTTAGAAAGGGTAAG
flgB2p −27/−28	GGGCGATTCCTTCGGAAAGGGTAAGGCGC
flgB2p −35	TAGAAAGGGTGGGGCGCGAAC
flgB2p −42/−43	GAAAGGGTAAGGCGGCAACAAAGAGGGATTTG
fliF2p −10	CCACATCCGTCCCGGATGGTCGGGC
fliF2p −35	GATTGTTGGGCCACATCCGTCA
fliI2p −10	TTGTTCGCGCCTCCCCCTTTCT
fliI2p −26/−27	TTAAGGGCGAGGCCTTTAGAAAG
fliI2p −35	CACCAAGGCTTCGGGGCGATTC
fliI2p −47/−48	CTCCCTCGATCGGTGCCACCAAGGCTTAAGGGC
fliL2p −10	GTCGATCTAGGGGCCGGATCCC
fliL2p −35	GATTGTTGGGCCACATCCGTCA

^a^The undelines bases correspond to the changes introduced in the wild type sequence

### Media and growth conditions

*R. sphaeroides* was grown in Sistrom’s minimal medium [30]. When indicated the strains were grown in Sistrom minimal medium in which succinic acid was reduced to 80 μM or replaced by 0.2% casamino acids. Heterotrophic growth was carried out at 30 °C in the dark with orbital shaking at 180 rpm. Photoheterotrophic growth on plates was achieved by incubation in a polycarbonate anaerobic jar containing a BD Gas Pack EZ anaerobe pouch system 8 (Becton, Dickinson and Company) and illuminated with two 75 W incandescent bulbs. Photoheterotrophic liquid cultures were grown in completely filled screw-cap tubes under continuous illumination. *Escherichia coli* was grown in LB medium [31] at 37 °C. When required, antibiotics were used at the following concentrations: for *R. sphaeroides*, kanamycin (25 μg/ml), tetracycline (1 μg/ml), spectinomycin (50 μg/ml). For *E. coli*, kanamycin (50 μg/ml), tetracycline (12 μg/ml) ampicillin (100 μg/ml), spectinomycin (50 μg/ml), chloramphenicol (20 μg/ml), gentamycin (30 μg/ml).

### Standard techniques of molecular biology

Routine genetic manipulations were performed as described elsewhere [31]. Restriction and modification enzymes were purchased from Thermo Fisher Scientific. PrimeSTAR HS from Takara Bio Inc. was used for DNA amplification.

### Isolation of the AR1 mutant strain

The intercistronic region between *flgB2* and *fliI2* as well as the 5′ region of each gene was replaced with the omega-Spc^R^ cartridge. For this a chromosomal region of 2250 bp was amplified using the oligonucleotides DinterB2For and DinterI2Rev. The PCR product encompassing from the end of *flgC2* to the end of *fliI2*, was cloned in pCR2.1-TOPO, digested with StuI and religated. The StuI digestion allowed the removal of two internal fragments, one of 392 and the other of 126 bp. The resulting linearized plasmid was self-joined to bring together the C-terminal half of *flgB2* with the C-terminal half of *fliI2*. This plasmid was purified, digested with StuI and ligated with the omega-Spc^R^ cartridge obtained from the pBOR plasmid. The complete fragment was then subcloned into pJQ200mp18 [32]. This plasmid was introduced to *E. coli* S17–1 and subsequently transferred to *R. sphaeroides* by conjugation [33]. Since pJQ200mp18 cannot replicate in *R. sphaeroides*, the double-recombination event was selected on LB agar plates in the presence of spectinomycin and 5% sucrose. The mutant was verified by PCR.

### Fusion of the flagellar promoters to the reporter gene *uidA*

The regulatory region of the *fla2* promoters for the genes *flgB2*, *fliI2*, *fliF2*, *fliL2* and *flgE2* was amplified by PCR using the oligonucleotides indicated in Table 2. The product was gel purified and cloned in pTZ19R for sequencing. Subsequently, each fragment was subcloned in pRK415/uidA. This plasmid enables to create a transcriptional fusion with the *uidA* gene that encodes for the β-glucuronidase. In pRK415/uidA, the *uidA*-*aadA* cassette was cloned in the BamHI site, the orientation of the fragment was selected in such a manner that the *uidA* gene can be transcribed from a promoter cloned as an EcoRI-SstI fragment.

### β-Glucuronidase assay

Cell-free extracts from exponential phase cultures grown photoheterotrophically were tested for β-glucuronidase activity following the previously reported protocol [34, 35]. As standard, a curve of different concentrations of 4-methyl-umbelliferone (Sigma-Aldrich) was used. Specific activities are expressed as μmol/min/mg of 4-methyl-umblliferone formed. Protein content was determined with a Bio-Rad protein assay kit, using bovine serum albumin as standard.

### Site directed mutagenesis

Mutagenesis was performed essentially following the method of Kunkel [36] with an uracil-containing single-stranded DNA as template and the appropriate oligonucleotides (Table 2). pTZ19R plasmids carrying *flgB2p*, *fliI2p*, *fliL2p*, and *fliI2p* were used as templates. The presence of the mutation was verified by sequencing.

### 5’-rapid amplification of cDNA ends (5´-RACE) analysis

Bacterial cells from cultures grown to mid-log phase in 0.2% casamino acids, were collected at 4 °C and immediately used to extract the complete pool of RNA using the RiboPure-Bacteria kit (ThermoFisher Scientific) according to the manufacturer’s instructions. Residual DNA in the samples was removed using DNaseI (Roche). To determine the 5′ end of the mRNA transcripts of *flgB2*, *fliI2*, *fliF2*, *fliL2*, and *flgE2*, the primers shown in Table 2 were used. The PCR products obtained from these reactions were cloned in pCR2.1-TOPO and sequenced.

### His6X-CtrA purification and antibody production

*ctrA* was amplified using the oligonucleotides ctrABADFwSac and ctrABADRvEco. The amplification product was cloned into pBAD/HisA (Invitrogen). This construction was introduced into Rosetta and a culture of this strain grown at mid-log phase was induced with 0.2% L-arabinose for 4 h at 37 °C. Cells were collected by centrifugation and resuspended in 1/100 of the original volume in phosphate buffered saline (PBS) 0.058 M Na_2_HPO_4_, 0.017 M NaH_2_PO_4_, 0.068 M NaCl, pH 7.4. The cell suspension was sonicated in an ice bath for five bursts of 10 s. Cell debris were removed by centrifugation and the supernatant was mixed with Ni-NTA-agarose beads (Quiagen) and incubated for 1 h on ice in the presence of 20 mM imidazole. The beads were loaded into a polypropylene column (1 ml of capacity) and washed with PBS/30 mM imidazole/200 mM NaCl. The protein was eluted using PBS containing 250 mM imidazole.

Six three weeks old BALB/C female mice, were immunized intraperitoneally with 20 μg of His6X-CtrA protein (for each mice) in incomplete Freund’s adjuvant, and 3 weeks later reimmunized. Two weeks later the mice were bled and antisera were obtained by low-speed centrifugation. The specificity of the anti-CtrA antibodies was tested by immunoblotting following standard protocols [37]; in this experiment, total cell extracts from AM1 and EA1 strains were used respectively as positive and negative controls.

### Chromatin immunoprecipitation (ChIP)

ChIP was carried out according to the protocol previously reported [33] with minor modifications as follows: 15 ml of a photoheterotrophic culture of *R. sphaeroides*, grown in Sistrom’s medium with 0.2% casamino acids, were transferred to a 125 ml Erlenmeyer flask when the OD_600_ reached 0.5. Immediately, 0.41 ml of 37% of freshly prepared formaldehyde was added and incubated for 10 min at 30 °C with slow shaking. After this time, the flasks were incubated on ice for 30 min. The cells were washed three times with PBS buffer and then resuspended in 250 μl of PBS with protease inhibitors (complete, EDTA-free protease inhibitor cocktail tablets from Roche). The sample was incubated at room temperature with lysozyme (80 μg/ml) for 15 min; after this time, 2.5 μl of Triton X-100 and 5 μl of EDTA 50 mM were added, and carefully mixed. After 10 min, the sample was sonicated 10 times for 8 s (30% duty cycle) on ice. Cell debris were removed by centrifugation at 10,000 x *g* for 5 min. The supernatant was transferred to a clean tube containing 100 μl of a mixture of Protein A Sepharose/DNA/BSA (100 μg of sonified herring sperm DNA/100 μg of BSA). The sample was incubated at 4 °C on a rocking platform shaker, for 2 h, and centrifuged for 3 min at 550 x *g*. The supernatant was transferred to a clean tube, and an aliquot of 20 μl was withdrawn and labeled as input. The remaining sample was mixed with 4 μl of anti-CtrA antibody and incubated overnight at 4 °C on a rocking platform shaker. The complexes were captured by adding 100 μl of Protein A-Sepharose/DNA/BSA and continuing incubation for 2 h. The immunoprecipitated material was washed twice in low, and high salt buffers (Low: 0.1% SDS, 1% Triton X-100, 2 mM EDTA, 150 mM NaCl, 20 mM Tris pH 8. High: 0.1% SDS, 1% Triton X-100, 2 mM EDTA, 500 mM NaCl, 20 mM Tris pH 8), twice in LiCl buffer (0.25 M LiCl, 1% Triton X-100, 1% deoxycholate, 1 mM EDTA, 10 mM Tris pH 8) and twice in TE buffer (10 mM Tris pH 8, 1 mM EDTA). The sample was resuspended in 500 μl of elution buffer (1% SDS, 0.1 M NaHCO_3,_ 300 mM NaCl) and incubated overnight at 65 °C to reverse the cross-linking. Input samples were also incubated overnight at 65 °C in elution buffer. The samples were treated for 30 min with 50 μg of RNase for 30 min at 37 °C, and subsequently with 120 μg of proteinase K for 30 min. DNA was purified using the QIAquick PCR purification kit. The procedure was carried out three times for AR1 and EA1 strains carrying the plasmid pRK_*flgB2p*::*uidA*-*aadA*; and twice for AR1 carrying pRK_*flgB2p* − 10::*uidA*-*aadA* or pRK_*flgB2p* − 35::*uidA*-*aadA*.

### Semiquantitative PCR

To assess the presence of the *flgB2-fliI2* region in the ChIP samples, we carried out a PCR reaction and evaluated the amount of product at different cycles of the reaction. The DNA products were analyzed by acrylamide gel electrophoresis and quantified using Image J software [38]. The data represent the average of three independent experiments.

### Identification of the CtrA binding sites

The DNA sequence containing the *fla2* genes was analyzed using a position weight matrix (PWM) built from the regulatory region of 54 genes identified to be controlled by CtrA in *C. crescentus* [6] with the tool matrix-scan included in the Regulatory Sequence Analysis Tools (RSAT) (http://embnet.ccg.unam.mx/rsat). Sequence alignments were carried out using MUSCLE [39].

## Results

### Construction of reporter plasmids containing selected *fla2* promoters

**Fig. 1:**
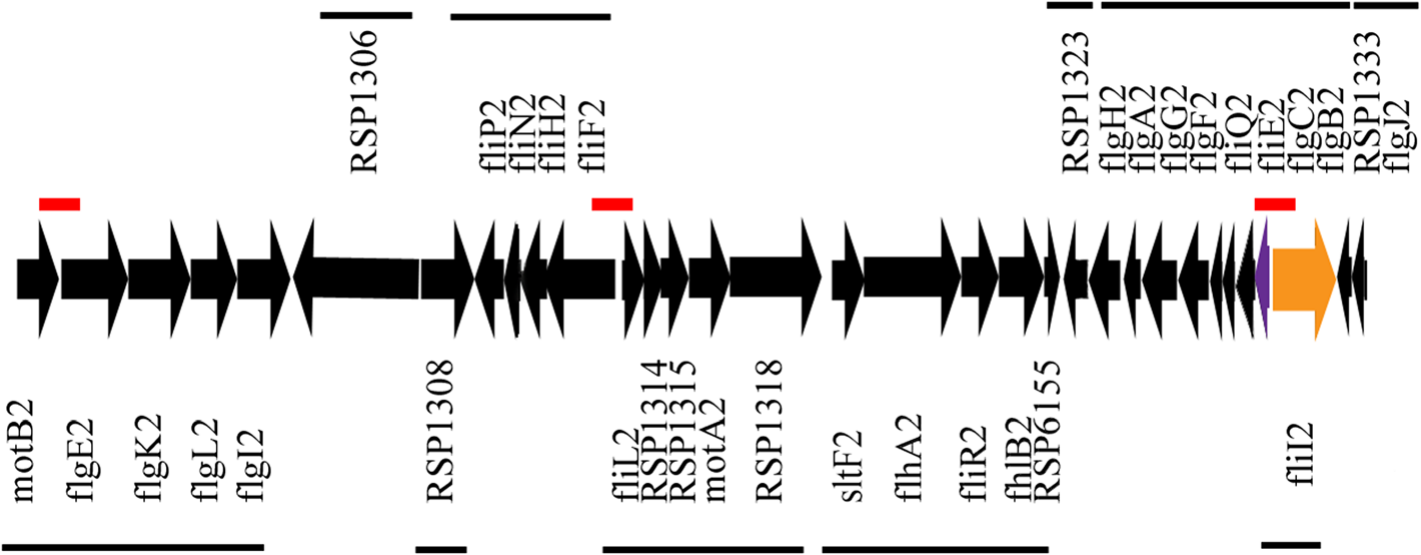
Cluster and operon arrangement of the *fla2* genes between *motB2* and *fliI2*. Arrows indicate the direction of transcription. The name of the genes that are transcribed from left to right are indicated under the arrows, and the genes transcribed in the opposite direction are indicated above the arrows. The red lines indicate the regulatory regions analyzed in this work. For an easy identification, the arrows representing *flgB2* and *fliI2* are colored in violet and orange, respectively. In the cluster, *motB2* encodes the motor protein B; *flgE2*, the hook protein; *flgK2* and *flgL2*, the hook associated proteins 1 and 3, respectively; *flgI2*, the P-ring protein; RSP_1316 a putative histidine kinase; RSP_1318 a putative response regulator; *fliP2* membrane component of the secretion apparatus; *fliN2*, a switch complex/secretion protein; *fliH2*, a soluble component of the secretion system; *fliF2*, the MS-ring protein; *fliL2*, a motor control protein; RSP_1414, RSP_1315, conserved hypothetical proteins; *motA*2, the stator protein A; RSP_1318, a conserved hypothetical protein; SltF, the flagellar soluble lytic transglycosylase (named before *flgJB2)*; *flhA2*, *fliR2*, *flhB2*, membrane components of the secretion apparatus; RSP_6155, conserved hypothetical protein; RSP_1323 similar to FliL; *flgH2*, L-ring protein; *flgA2*, chaperone for P-ring formation; *flgG2*, distal rod protein; *flgF2*, rod protein; *fliQ*, membrane component of the secretion apparatus; *fliE*, periplasmic protein related with the secretion apparatus; *flgC2*, *flgB2*, proximal rod proteins; *fliI2* cytoplasmic component of the secretion system, ATPase component; RSP_1333 putative secretion chaperone; *flgJ2*, cap rod protein

The *R. sphaeroides* WS8N genome consists of two chromosomes and two plasmids. Most of the *fla2* genes are clustered in a region of approximately 32.3 Kb localized in chromosome I (Fig. 1) and the gene encoding flagellin of the Fla2 flagellum is in plasmid A [11, 40].

The *fla2* chromosomal region was analyzed searching for putative CtrA binding sites as described in Materials and Methods. Using a threshold of 5, the search identified 57 hits, many of which were within coding regions. A threshold of 5.5 identified 34 hits, and the best hits included the regulatory regions of *motB2*, *flgE2*, *motA2*, as well as the intercistronic regions containing the divergent promoters of *fliF2*-*fliL2*, *flgB2*-*fliI2* and *flgJ2*-*fliK2* (Additional file 1). Given the palindromic nature of the CtrA binding site, frequently two hits were computed for each promoter; we also noted that in a single regulatory region several hits were occasionally found (for instance upstream of *motA2*). Eleven hits mapped within coding regions, suggesting a high rate of false negatives.

To evaluate the functional relevance of some of the identified sites, we cloned the intercistronic regions located between *fliL2*-*fliF2*, and *flgB2*-*fliI2* in a plasmid that carries the reporter gene *uidA* (pRK415/uidA), in order to create a transcriptional fusion between these promoters and the *uidA* gene, which encodes for the enzyme β-glucuronidase. To test the activity of the promoters *fliL2p*, *fliF2p*, *flgB2p* and *fliI2p*, these regions were cloned in both orientations regarding *uidA*. These constructions were introduced to the AM1 mutant strain (for simplicity this strain will be named as wild type from now on) and its *ΔctrA* derivative (EA1 strain). The amount of β-glucuronidase produced by each strain was determined.

It was observed that all these plasmids promoted an elevated synthesis of β-glucuronidase in the AM1 (WT) strain, but not in the EA1 (Δ*ctrA*) strain (Table 3). This suggests that each one of these regions contain a functional CtrA-dependent promoter. The region upstream of *flgE2* was also cloned into pRK415/uidA, in order to test a region that does not contain a divergent promoter. β-glucuronidase activity promoted by *flgE2p* was also dependent on the presence of CtrA (Table 3).

**Table 3:** β-glucuronidase activity promoted by the regulatory regions of *flgE2p*, *fliF2p, flgB2p*, *fliL2p*, and *fliI2p*^a^

	WT	*ΔctrA*
*flgE2p*	148 ± 32	1.4 ± 0.36
*fliF2p*	166 ± 27	1.0 ± 0.24
*flgB2p*	125 ± 24	0.52 ± 0.12
*flgL2p*	204 ± 36	0.28 ± 0.06
*fliI2p*	99 ± 19	0.37 ± 0.09

^a^The plasmids pRK415/uidA carrying the indicated regulatory regions were introduced to AM1 (WT) and EA1 *(ΔctrA*) and the amount of β-glucuronidase was determined. Activity is expressed as μmol of 4-methyl-umblliferone/min/mg of protein. The values represent an average of three independent experiments

To validate our plasmid system, we cloned the *fliQ* promoter from *C. crescentus*, previously known to be activated by CtrA, into pRK415/uidA and it was introduced to the wild-type (AM1) and EA1 strains. A high level of β-glucuronidase was detected in AM1 but not in EA1 (data not shown).

### Determination of the transcriptional start site of *fliL2p*, *fliF2p*, *flgB2p*, *fliI2p* and *flgE2p*

**Fig. 2:**
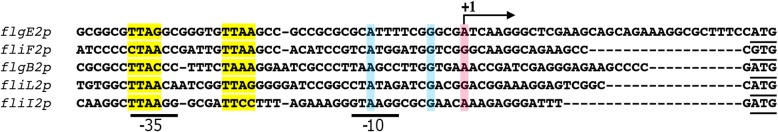
Transcriptional start sites of the *flgE2*, *fliF2*, *flgB2*, *fliL2* and *fliI2* promoters The transcriptional start site (+ 1), is indicated by a bent arrow and the nucleotide is shaded in pink. The conserved nucleotides A and G at − 11 and − 4 positions, respectively are shaded in blue. The nucleotides matching with the consensus CtrA-binding site are highlighted in yellow. The translation codons for FlgE2, FliF2, FlgB2, FliL2 and FliI2, are underlined

To further characterize *fliL2p*, *fliF2p*, *flgB2p*, *fliI2p* and *flgE2p*, we proceeded to determine the transcriptional start site (TSS) by 5’-RACE. The TSS was established by sequencing five independent clones from each sample. For these promoters, the initiation nucleotide was always a purine (Fig. 2). The regions upstream of the TSS were aligned using MUSCLE and also by visual inspection; from this alignment it was possible to detect a sequence similar to the CtrA-binding site (yellow shaded in Fig. 2). The distance between the TSS and the final boundary of the putative CtrA-binding site is 23 or 24 nt, which is in agreement with the findings made in *C. crescentus*. In this bacterium it was observed that the CtrA-binding site is located near the − 35 promoter region in CtrA-activated promoters [18, 20]. It should be stressed that the putative CtrA-binding sites identified from this sequence alignment, concur with those identified bioinformatically using a threshold of 5.5, except for the divergent promoters *flgB2p* and *fliI2p*.

From the alignment (Fig. 2), we also observed an invariant A at the − 11 position, a G at − 4 and, a variable distance between the TSS and the start codon, ranging from 33 to 12 bp. Under the idea that the − 11 position could be a part of the − 10 promoter region, we presume that the identity of the − 11 position could be important for transcription.

As can be observed in Fig. 2, a gap was introduced between the left and right conserved elements of the CtrA-binding site for *flgB2p* and *fliI2p*. Therefore, these sites do not conform to the consensus CtrA-binding site, and in consequence they were not found by the algorithm in the initial analysis. For *flgB2p*, the algorithm predicted two different sites. The first has a score of 8.3 and overlaps the TSS. The other starts one nucleotide upstream of the highlighted sequence in Fig. 2 but has several differences from the consensus and a score just above the threshold (5.9), which contrasts with the high score observed for the predicted sites upstream of *fliF2p*, *fliL2p* or *flgE2p* (i.e., 9.7, 9.3 and 10.3, respectively). For *fliI2p*, the algorithm predicted one CtrA-binding site (score 7.1), which starts upstream of the sequence shown in Fig. 2, and overlaps with the highlighted sequence in Fig. 2, in such a manner that the right element of this site is the left element highlighted in Fig. 2. To clarify the relevance of these putative CtrA binding sites, additional experiments were carried out.

### Site directed mutagenesis of the *fla2* promoters

**Fig. 3:**
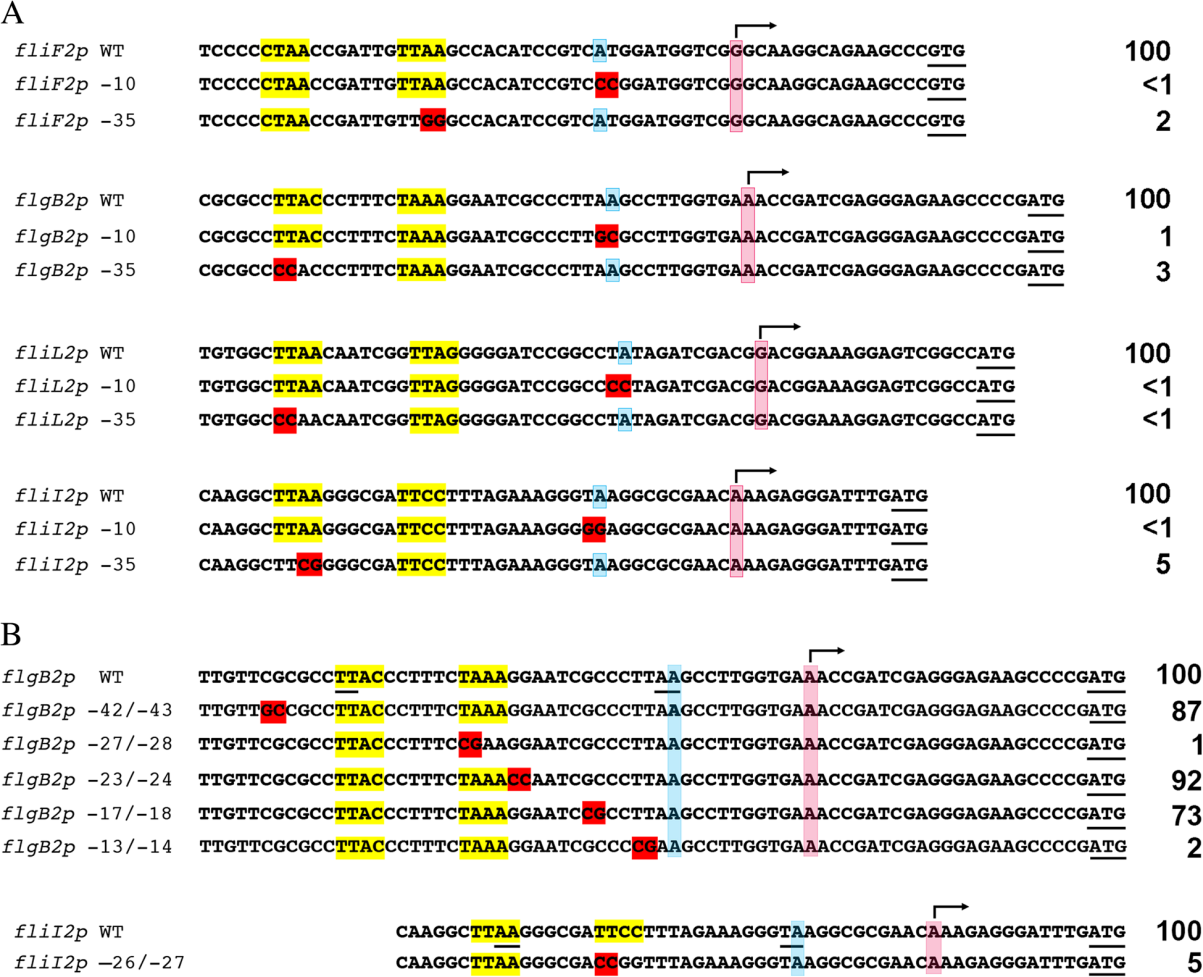
Activity of the wild-type and mutant versions of *fliF2p*, *flgB2p*, *fliL2p* and *fliI2p* in the AM1 strain. The amount of β-glucuronidase shown at the right, is expressed as percentage of the activity determined for the wild-type promoter. The values are the average of three independent experiments, SD was less than 25%. The nucleotides shaded in red represent the mutated bases regarding the wild-type promoter. The transcriptional start sites, are indicated by a bent arrow and the nucleotide is shaded in pink. The conserved A at − 11 is shaded in blue. The nucleotides matching with the consensus CtrA-binding site are highlighted in yellow. The translational codon is underlined. **a**) Activity of the wild-type and mutant promoters with changes affecting the A − 11 and the CtrA-binding site. **b**) Activity of the wild-type and mutant promoters affecting different regions in *flgB2p* and *fliI2p*. The underlined nucleotides upstream the transcriptional start site, indicate the mutagenized positions in the constructions tested in panel (**a**)

To gain insight on the role of the sequences identified from the alignments of *flgB2p*, *fliI2p*, *fliF2p* and *fliL2p* such as the putative CtrA binding site or the putative − 10 promoter region, we proceeded to mutagenize the nucleotides shown in Fig. 3a. For each promoter we changed two bases of the proposed CtrA-binding site and two bases of the putative − 10 promoter region.

The promoter regions carrying these changes were cloned in pRK415/uidA and introduced to AM1. The amount of β-glucuronidase present in total cell extracts was determined. We noticed a strong reduction in the enzymatic activity when these changes were introduced (Fig. 3a), suggesting that these regions could indeed represent the functional CtrA binding site and the − 10 promoter region. To support this notion, we decided to further analyze *flgB2p* introducing additional changes along the regulatory region (Fig. 3b). The plasmids carrying these changes were introduced to AM1 and the amount of β-glucuronidase was determined. We noticed a reduction in the amount of β-glucuronidase when the positions immediately upstream of the − 11 position (− 13/− 14), and the right element of the CtrA-binding site (− 27/− 28) were changed (Fig. 3b). Mutations at positions − 17/− 18, − 23/− 24, and − 42/− 43 did not severely affect the expression of the reporter gene. These results support the notion that specific positions at this promoter region are indeed relevant to achieve transcription whereas others do not make a significant contribution. Therefore, the region around the − 11 position and the proposed CtrA binding site, including the left and right elements, are key for the expression of *flgB2p*.

For *fliI2p*, the right element of the proposed CtrA binding site, was also mutagenized, and this change strongly reduced the amount of β-glucuronidase as compared with the wild type promoter (Fig. 3b), indicating that the proposed sequence could represent the actual CtrA-binding site. As mentioned in the previous section, a different site was predicted bioinformatically for this promoter. The right element of this putative site is in fact the left element highlighted in the alignment shown in Figs. 2 and 3. To obtain evidence of the contribution of this putative site, we mutagenized the − 47/− 48 positions of *fliI2p*, which must represent the left element, but did not detect a reduction in the amount of β-glucuronidase when this mutant promoter was tested in pRK415/uidA (data not shown). These results lead us to the conclusion that the functional site for CtrA binding must be the sequences highlighted in yellow in Fig. 2.

**Fig. 4:**
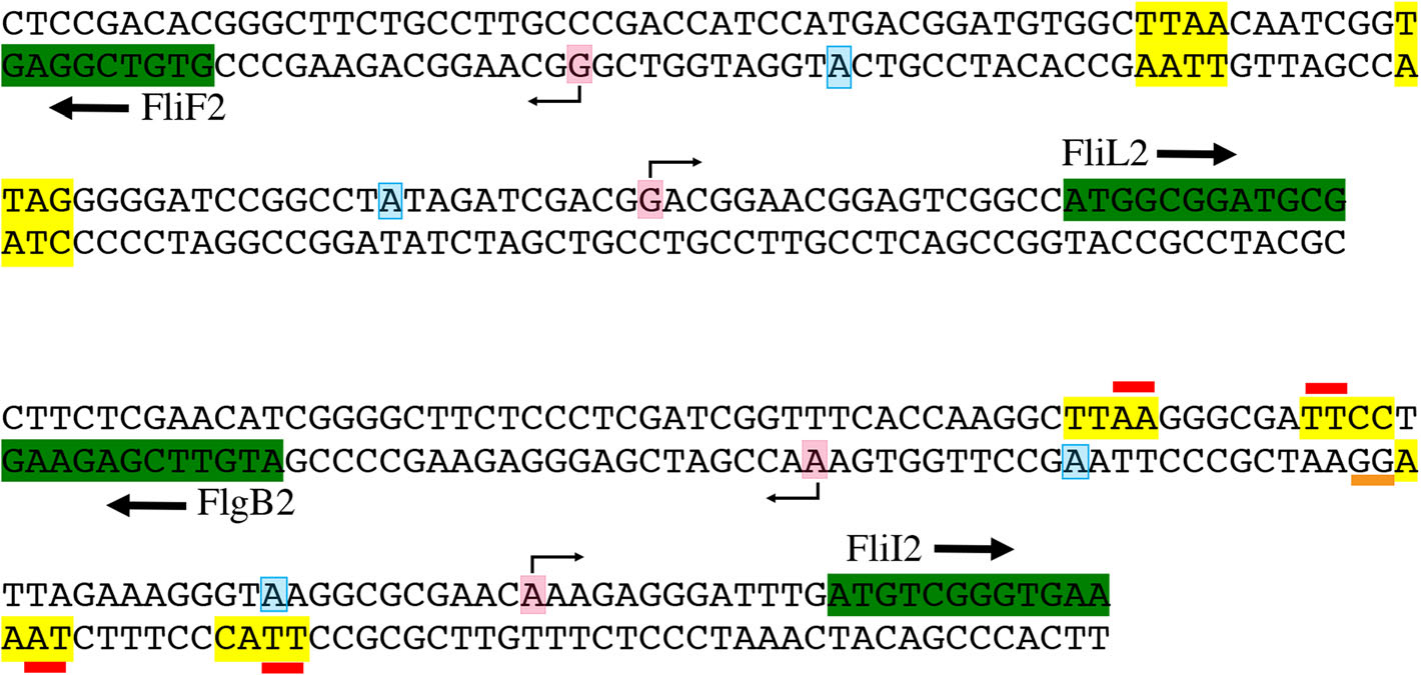
Architecture of the divergent promoters *fliF2p*-*fliL2p* and *flgB2p*-*fliIp*. The complete intercistronic region between these genes is shown. The coding region for FliF2, FliL2, FlgB2 and FliI2, represented by the initiation and 3 or 4 additional codons, is highlighted in green. The nucleotides matching the consensus CtrA-binding site are highlighted in yellow. For the intercistronic region between *fliF2* and *fliL2*, the CtrA binding site is the same for both promoters; therefore, the highlighted nucleotides are on both strands. For the intercistronic region between *flgB2* and *fliI2*, the CtrA binding site for *fliI2p* is highlighted on the top strand and the CtrA binding site for *flgB2p* is highlighted on the bottom strand. For reference the conserved A at the − 11 position is shaded in blue (on the top strand for *fliI2p* and on the bottom strand for *flgB2p*). The transcriptional start site is indicated by a bent arrow and shaded in pink. For the *flgB2*-*fliI2* intercistronic region, a red bar below the sequence indicates the changes that the reduced the expression of *flgB2p*. A red bar above the sequence indicates the changes that reduced the expression of *fliI2p*. The orange bar below the sequence indicates the mutagenized position that did not affect the expression of *flgB2p* (− 23/− 24) but is located in the CtrA-binding site for *fliI2p* (highlighted in yellow in the top strand)

As mentioned before, *flgB2* and *fliI2* are transcribed from divergent promoters as well as *fliF2* and *fliL2*; nonetheless, from the above results we realized that the architecture of these promoter regions is different. Figure 4 illustrates how a single CtrA binding site is sufficient for activation of *flgF2p* and *fliL2p*, whereas *flgB2p* and *fliI2p* are activated from independent CtrA binding sites.

### Binding of CtrA to *flgB2p* and *fliI2p* promoters

Attempts to detect binding of His6X-CtrA to different *fla2* promoters by gel electrophoresis mobility shift assay (EMSA) and DNAse I footprinting were unsuccessful; therefore, we decided to undertake an in vivo approach using chromatin immunoprecipitation (ChIP) in order to reveal the binding of CtrA to the *fla2* promoters. For these experiments we used the plasmid pRK415/uidA carrying *flgB2p* in the AR1 strain, in which the chromosomal region corresponding to *flgB2p* and *fliI2p* was deleted.

**Fig. 5:**
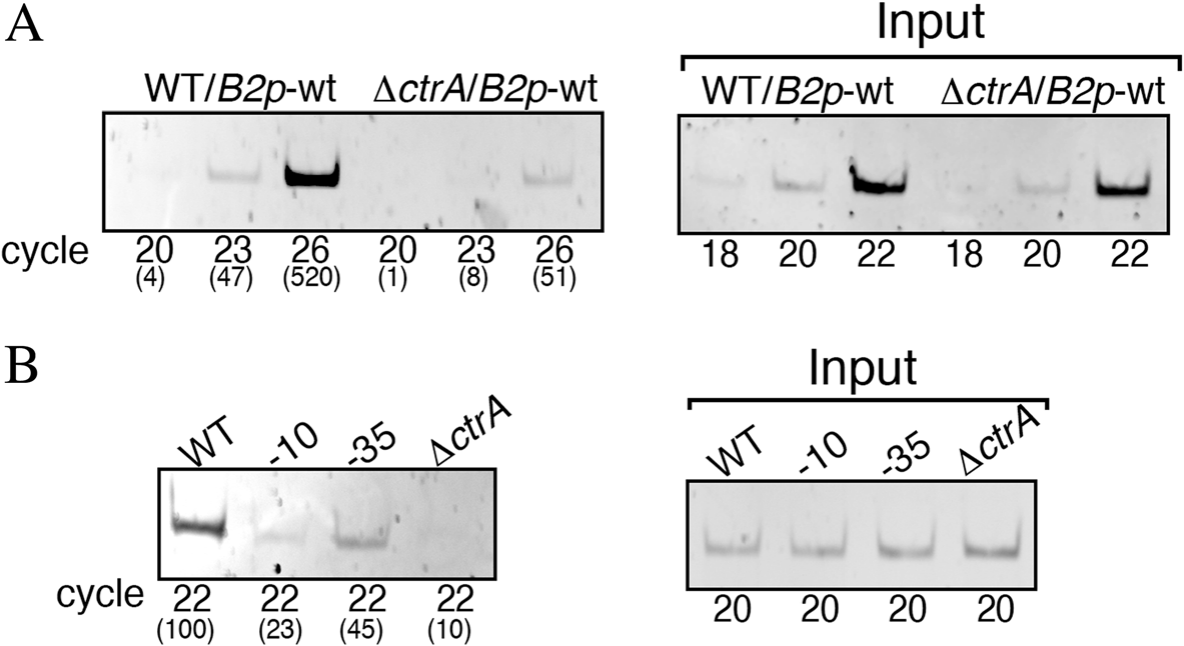
Binding of CtrA to the *flgB2*-*fliI2* regulatory region. DNA material immunoprecipitated with α-CtrA antibodies, was amplified using the flgBI2 and flgBI5 oligonucleotides. Samples were taken at different cycles and analyzed by agarose gel electrophoresis. Panel **a** and **b** show one representative experiment out of three independent assays. **a**) Samples of AM1 (WT) and EA1 (*ΔctrA*) carrying pRK_*flgB2p*::*uidA*-*aadA* (*B2p*-wt) were amplified during the number of cycles indicated below. The numbers in parenthesis represent the value obtained from the densitometric analysis. Input, shows the amplification products from an aliquot taken from each sample before ChIP (total chromatin input DNA). **b**) Samples of AR1 carrying pRK_*flgB2p*::*uidA*-*aadA* (WT) or the mutant versions pRK_*flgB2p* − 10::*uidA*-*aadA* (− 10) and pRK_*flgB2p* − 35::*uidA*-*aadA* (− 35), and EA1 carrying pRK_*flgB2p*::*uidA*-*aadA* (Δ*ctrA*). The values in parenthesis represent the percentage of product relative to the amount detected for the wild type. Input shows the amplification products from an aliquot taken from each sample before ChIP

The amount of *flgB2p* immunoprecipited by α-CtrA antibodies was detected by semi-quantitative PCR. A strong increase of the PCR product was observed in the sample obtained from AR1 as compared with that obtained from EA1 *(ΔctrA*), indicating that CtrA binds to this region (Fig. 5a).

We also analyzed the amount of *flgB2p* precipitated by α-CtrA antibodies using the AR1 strain transformed with the plasmids carrying *flgB2p* − 10 and *flgB2p* − 35 (constructions shown in Fig. 3a). Given the architecture of *flgB2p* and *fliI2p* it should be stressed that the change at the − 10 region of *flgB2p* affects the CtrA-binding site of *fliI2p* (Fig. 4), so a reduction in the amount of product can be expected. From these experiments we observed a perceptible reduction in the amount of DNA that was immunoprecipitated by α-CtrA antibodies when the putative CtrA-binding sites were mutagenized, supporting the idea that these sites are involved in the binding of CtrA (Fig. 5b).

### Analysis of the intercistronic region *flgB*-*fliI* in other α-proteobacteria

**Fig. 6:**
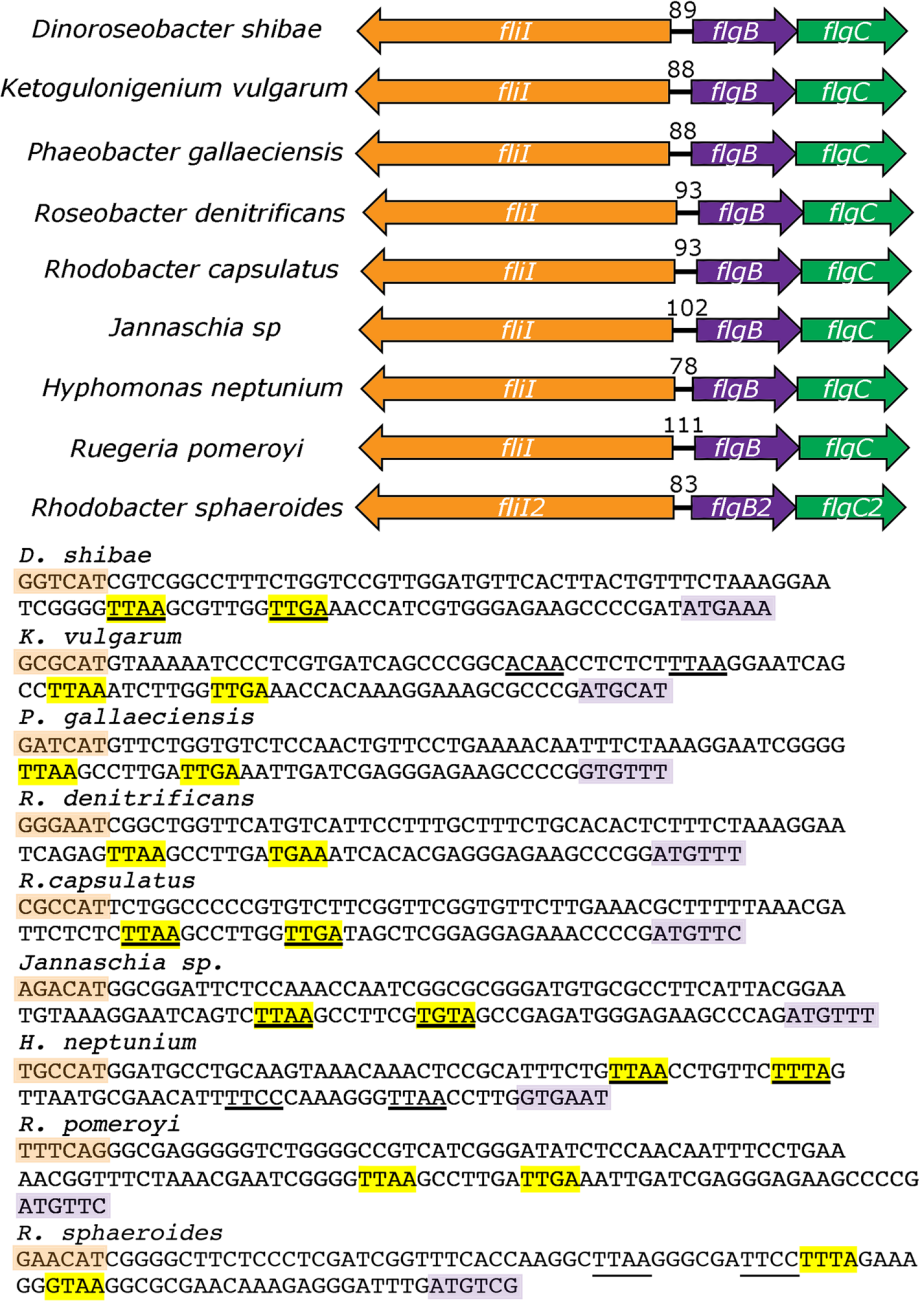
CtrA binding sites for the *fliI*-*flgB* divergent promoters from different species. Intercistronic regions between *flgB* and *fliI* of six different species of *Rhodobacteraceae* and one species of the *Hyphomonadaceae* family. These genes show a similar arrangement as that observed for *flgB2* and *fliI2* of *R. sphaeroides*. The *fliI* gene is represented in orange and *flgB* in violet. Below, the complete intercistronic region is shown and the sequences predicted as possible CtrA binding sites are indicated as follows: highlighted in yellow for the reverse strand, and underlined for the direct strand. For *Hyphomonas neptunium* two different sites are underlined given that the highest score (4) corresponds to a predicted site that is five nucleotides upstream the initiation codon of FlgB; the other possible site has a lower score (3.8) but from this localization it is likely that CtrA could activate the RNA polymerase

The *flgB2* and *fliI2* genes are syntenic in several **α-**proteobacteria. We analyzed the intercistronic region between these genes for seven species of *Rhodobacteraceae* and one from the *Hyphomonadaceae* family, order *Rhodobacterales*. These regulatory regions are shown in Fig. 6, and it is evident that there is a limited space for the promoters and the CtrA-binding sites. For *Rhodobacter capsulatus*, *Dinoreoseobacter shibae* and *Ruegeria pomeroyi*, it has been established that the expression of the flagellar genes is dependent on CtrA [29, 41, 42] and given that in the other species it is possible to find the presence of the gene encoding the histidine kinase *cckA*, the phosphotransferase *chpT* along with *ctrA*, an educated guess allows us to presume that in these bacteria the flagellar genes are also controlled by CtrA. Using the PWM previously published for *C. crescentus* [17], we searched for the CtrA-binding sites in these sequences using a threshold of 3. From this analysis we noticed that for some species it was not possible to find putative CtrA binding sites with scores above 3.5, and in other cases only one site with a high score was found (Fig. 6). From this, it seems possible that a single CtrA binding site could be used to activate these promoters in *Dinoroseobacter shibae* and *Jannaschia sp*, whereas two CtrA-binding sites seem to be required for activation in *Ketogulonigenium vulgarum*. Nonetheless, from this analysis it emerges that more than one type of architecture is possible.

## Discussion

We have previously shown that in *R. sphaeroides* the expression of the *fla2* genes is controlled by CtrA [15]. In this work we present evidence indicating that CtrA binding directly activates transcription of these genes. From our results we inferred that the binding site for this transcription factor should be similar to the one already reported for *C. crescentus*, given that the *fliQ* promoter from this microorganism was able to transcribe the reporter gene *uidA* in a CtrA-dependent fashion in *R. sphaeroides*. Therefore, we analyzed bioinformatically the *fla2* region for CtrA binding sites using the PWM obtained from studies in *C. crescentus* [17]; several sites were identified and its functionality was tested by site directed mutagenesis. This analysis together with the identification of the TSS of several *fla2* promoters allows us to define the control region of these genes. We observed that they contain an invariant A at the − 11 position, and a putative CtrA binding site near the − 35. Adenine at the − 11 position could represent a relevant contact for the RNA polymerase associated with the σ^70^ factor. In line with this idea, it has been proposed that this nucleotide is flipped out during the open complex formation and is specifically recognized by several amino acids in the sigma-2 domain of σ^70^[43]. In addition, we determined that a purine is located at the initiation transcription site in these four promoters, which is in agreement with previous observations in other bacterial promoters [44–46]. Therefore, several common features previously known to be relevant for transcription initiation are present in these promoters.

We centered our analysis in the divergent promoters *fliL2p*-*fliF2p*, and *flgB2p*-*fliI2p* given that transcription from divergent promoters that are dependent on positive control could reveal additional features to those already known for non- divergent promoters. It is known that divergent promoters not only show a reduced space for housing the RNA polymerase and other transcription factors, but may also be affected by changes in the local superhelical density generated by the adjacent transcribing RNA complexes [47–49].

We observed that, regardless that the intercistronic region of *flgB2* and *fliI2* is only 47 bp counting from the transcription start sites (TSS), there are two independent CtrA-binding sites in this region. This is supported by the fact that site-directed mutagenesis of the CtrA binding site of *flgB2p* (in the left or right element corresponding to *flgB2*–35 and − 27/− 28 constructions, see Figs. 3 and 4) provoked a decrease of the activity of the reporter gene; however, mutagenesis of the − 23/− 24 positions of *flgB2p* did not affect the expression of the reporter gene, but did affect the functional CtrA binding site of *fliI2p* (see Fig. 4)*.* This suggests that binding of CtrA to activate *fliI2p* did not affect activation of *flgB2p*, at least when these promoters are measured from a plasmid.

It should be stressed that the proposed CtrA-binding sites for these promoters (i.e. *flgB2p* and *fliI2p*) do not conform to the consensus that has been previously reported for other bacteria, given that for *flgB2p* and *fliI2p*, the left and right elements of the CtrA binding site are located only 6 bp apart. As explained in the results section, for *flgB2p*, the other possible sites for CtrA binding were unlikely to be functional, given that one overlapped with the TSS, and the other showed a very low score. For *fliI2p* the best CtrA binding site that was identified bioinformatically was not supported by the experiments reported in this work. Therefore, we presume that for these promoters CtrA is able to activate transcription from atypical binding sites. A possible scenario could be that a very short intercistronic region with divergent promoters could be particularly affected by negative supercoiling (undertwisting) of the DNA. This conformation would be due to the activity of the divergent transcription from these promoters, enabling a better recognition of the CtrA binding site regardless of the fact that the left and right elements are positioned only 6 bp apart. A similar spacing between the left and right TTAA elements of the CtrA binding site has been described for *Magnetospirillum magneticum* [21]. An atypical spacing between these elements was also noted for the *ctrA* binding sites of the *ctrA* P1 and P2 promoters of *C. crescentus.* In this case the P1 promoter that is negatively controlled by CtrA, could have the ungapped variant for CtrA binding; however, for the CtrA-activated P2 promoter, the contribution of the left and right elements spaced by 6 bp was verified by an electrophoretic mobility shift assays [50].

A different situation was observed for activation of *fliF2p* and *fliL2p*, given that it appears that only one CtrA binding site is required for activation of these other divergent promoters. These results suggest that the CtrA binding site and both promoters must be properly placed on the same face of the DNA helix. In this regard, we identified a single CtrA binding site between the divergent promoters *fliI* and *flgB* in several species of α-proteobacteria, suggesting that the presence of a single CtrA binding site to active divergent promoters could be a common feature. Alternatively, it is possible that the PWM does not represent the actual CtrA binding site in the regulatory regions of these bacteria, in which case other CtrA binding sites would remain to be identified.

In *C. crescentus* it has been reported that the divergent promoters *flgBp* and *fliOp*, which are located 144 bp apart, share a CtrA-binding site. In this case, *flgBp* is repressed by CtrA and *fliOp* is activated when CtrA binds to this site [27]. This difference could be the result of a different evolution of the flagellar transcriptional hierarchies. For instance, in *C*. *cresentus* the periplasmic components of the flagellum (most of the genes encoding the rod proteins, and FlgI and FlgH that form the L and P rings, respectively) are encoded by genes that are dependent on RpoN and the activator protein FlbD [9]. In *R*. *sphaeroides* none of its four different RpoN proteins [51] is involved in the expression of the *fla2* genes (unpublished results). Moreover, the gene encoding FlbD is absent in the species of *Rhodobacteraceae* shown in Fig. 6; nevertheless, other proteins of this family of activators can be identified. This information suggests that in *R. sphaeroides* and other species of this genus, the early constituents of the flagellar structure including the periplasmic components are encoded by genes that could be directly activated by CtrA.

## Conclusions

In this work we show that CtrA directly activates the expression of the *fla2* genes of *R*. *sphaeroides*. The identification of the transcriptional start site for several *fla2* promoters allowed us to identify several conserved features such as a conserved A at the − 11 position and a purine at the initiation start site. The CtrA binding sites were tested by site directed mutagenesis and it was found that CtrA activates the expression of the divergent promoters *fliL2p*-*fliFp2* using a single binding site, whereas for the divergent promoters *flgB2p*-*fliI2p* CtrA activates transcription from two independent sites. In addition, we found that the CtrA binding site could also be functional when the left and right elements of the full motif are 6 bp apart.

## Additional file

Additional file 1: CtrA-binding sites predicted in the *fla2* cluster of *Rhodobacter sphaeroides* using the PWM reported for *Caulobacter crescentus.* Hits found by RSAT in the *fla2* cluster of *R. sphaeroides*. (XLSX 40 kb)

## Abbreviations

5’-RACE: 5’-Rapid amplification of cDNA ends
Ap: Ampicillin
BSA: Bovine serum albumin
ChIP: Chromatin immunoprecipitation
Cm: Chloramphenicol
EDTA: Ethylenediaminetetraacetic acid
EMSA: Gel electrophoresis mobility shift assay
Gm: Gentamycin
LB: Luria broth
MEME: Multiple EM (expectation-maximization algorithm) for Motif Elicitation
min: Minutes
MUSCLE: Multiple Sequence Comparison by Log-Expectation
Nal: Nalidixic acid
Ni-NTA: Nickel-nitrilotriacetic acid
OD600: Optical density measured at 600 nm
PBS: Phosphate buffered saline
PCR: Polymerase chain reaction
PWM: Position weight matrix
rpm: revolutions per minute
RSAT: Regulatory Sequence Analysis Tools
SD: Standard deviation
SDS: Sodium dodecyl sulfate
Spc: Spectinomycin
TCS: Two component system(s)
Tris: Tris(hydroxymethyl)aminomethane.
TSS: Transcriptional start site
